# Telemonitoring of Patients With Chronic Traumatic Brain Injury: A Pilot Study

**DOI:** 10.3389/fneur.2021.598777

**Published:** 2021-04-01

**Authors:** Maria Girolama Raso, Francesco Arcuri, Stefano Liperoti, Luca Mercurio, Aldo Mauro, Francesco Cusato, Lidia Romania, Sebastiano Serra, Loris Pignolo, Paolo Tonin, Antonio Cerasa

**Affiliations:** ^1^Sant'Anna Institute, Crotone, Italy; ^2^Institute for Biomedical Research and Innovation, National Research Council, Palermo, Italy

**Keywords:** telerehabilitation, traumatic brain injury, medical complications, healthcare costs, coma recovery scale revised

## Abstract

Telehealth systems have shown success in the remote management of several neurological disorders, but there is a paucity of evidence in disorders of consciousness (DOC). In this study, we explore the effectiveness of a new telemonitoring system, for monitoring Vegetative State (VS) and Minimally Conscious State (MCS) patients. This was a prospective, mono-center randomized controlled study. We included only traumatic brain injury (TBI) patients who required long-term motor/cognitive assistance having a stable clinical condition. We examined their clinical evolution over ~4 years of the follow-up period. Twenty-two TBI patients were enrolled and equally divided into two groups: one telemonitored at home with our service and the second admitted to a standard long-stay hospitalization (LSH) program. Patients enrolled in the telehealth service (age: 49.9 ± 20.4; 45% female; diagnosis: 36% VS/64% MCS) were demographically and clinically-matched with those admitted to the LSH program (age: 55.1 ± 15; 18% female; diagnosis: 54% VS/46% MCS). Thirty-six percent of patients in the LSH program died before completing follow up evaluation with respect to 18% of death in the group of TBI patients telemonitored at home. At follow-up, patients in LSH and telemonitoring groups showed similar clinical progression, as measured by CRS-r, NCS, WHIM, and LCF scales, as well as by the number of medical complications (i.e., bedsores, infections). Finally, we estimated the total daily cost per patient. Severe TBI patients enrolled in the conventional LSH program cost 262€ every single day, whereas the cost per patient in the telehealth service resulted to be less expensive (93€). Here, we highlight that our telehealth monitoring service is as efficacious as in-person usual care to manage a severe neurological disorder such as TBI in a cost-effective way.

## Introduction

Traumatic brain injury (TBI) is one of the leading causes of death and disability worldwide. Annually, over 2 million incidents are causing traumatic brain injury (TBI) and while research is continually accumulating to better understand the trajectory of clinical course, treatment options lag (1). Recovery from TBI is a complex process and severe brain injuries commonly result in a wide range of disorders of consciousness (DOC). This condition is characterized by high heterogeneity in clinical phenotypes and, mainly, in prognostic models (2–4) that contributed to disappointing results in several clinical trials (5).

Functional recovery following TBI usually reaches its peak at around 6 months and begins to decline as soon as 1 year after the neurological event (6). Generally, the vast majority of patients receive high-quality care and support in intensive neurorehabilitation unit (IRU) and are discharged successfully back to their communities. However, a significant minority of patients often continue to suffer from limited independence and face very long stays in rehabilitation wards that are far from their homes and families (7). Long-term hospitalization (LSH) is generally required for unstable TBI patients, although there is pressure to manage patients outside of the hospital in order to reduce costly hospital resources. Thus, there is a need for new post-discharge programs that may support families in caregiving, fostering, at the same time, better functional status and reducing healthcare service access, hospitalization, and costs.

One promising avenue to answer this need is telerehabilitation. As recently stated by the World Federation for NeuroRehabilitation (http://wfnr.co.uk/), telerehabilitation can be divided into different levels: (a) from the basic intervention of telecounselling and telecare; (b) passing from telemonitoring (with physiological data recorded by wearable devices); (c) until to teletherapy (where patients underwent specific treatments for improving clinical status). Overall, after several years of studies, telerehabilitation is considered an important tool for improving health and quality of life in neurological patients living in nursing homes, and potentially reducing healthcare hospitalization, service access, costs, finally reducing the caregivers' burden (8, 9).

In older adults with multiple chronic conditions, Takahashi et al. (10) demonstrated that telemonitoring was effective in reducing hospitalizations and physician visits when compared with usual care. In patients with Parkinson's disease (PD), it has been shown that telerehabilitation is feasible, well-received by patients and caregivers, even in more severe disease states (11). Outcomes are also similar between telerehabilitation and usual in-person care. In particular, Beck et al. (12) revealed no worsening of clinical outcomes in PD patients, including a number of emergency room visits, hospitalizations or level of caregiver burden in patients undergoing a telerehabilitation intervention with respect to patients enrolled in a usual care group requiring hospitalization. Finally, the experiences of AD-related telerehabilitation programs have demonstrated several advantages mainly in increasing the number of physician visits, in reducing the distance traveled by caregivers and in time spent traveling (11, 13). Considering long-term outcomes, Kim et al. (14) compared individuals who received their dementia care through video-based visits conducted at a clinic and who received their dementia care at the university hospital. They found no significant difference in cognitive decline (measured with MMSE score) between the two groups over 2 years of follow-up.

Despite telehealth systems have shown success in remote management of several neurological disorders, there is a paucity of evidence in DOC. The aim of this study is to determine the effectiveness of a new telehealth follow-up program for patients with TBI, which ensures h24 high level of assistance with multi-parametric vital sign monitoring, and periodic neurological and neuropsychological teleconsulting. We specifically examine the efficacy of this management strategy, by comparing long-term clinical outcomes of chronic TBI patients with respect to another demographically and clinically matched group of TBI patients admitted to a usual LSH program.

## Materials and Methods

### Participants

The study was realized on patients with severe brain injuries who required long-term motor/cognitive assistance, consecutively enrolled at the time of their transfer from the IRU to LSH period, within the S. Anna Institute (Crotone, Italy). The evaluation for enrollment in this study was performed at admission in long-term care. The inclusion criteria were: (1) diagnosis of acquired TBI according to neuroradiological and clinical assessments; (2) patients having a stable clinical condition; (3) absence of infections; (4) age range 18–75 years; (5) availability of receiving in-home rehabilitation service; and (6) availability of a home internet connection. Exclusion criteria were: (1) cardiorespiratory instability; (2) high-risk of spontaneous fractures; (3) presence of other severe pathologies influencing the outcome; (4) refusal by the caregiver of the patient's home transfer.

The study was approved by the Ethical Committee of the Central Area Regione Calabria in Catanzaro, according to the Helsinki Declaration. The surrogate decision-makers of the patients enrolled in the study provided their written informed consent. The original forms were collected and stored. All the experimental procedures were conducted according to the policies and ethical principles of the Declaration of Helsinki.

### Design and Procedure

This was a mono-center randomized controlled study, evaluating the clinical evolution of severe TBI patients after ~4 years intervals from admission in two different long-term care facilities (LSH or telemonitoring).

From January 2012 and December 2015 all patients admitted in LSH after discharge from IRU were evaluated to identify the subjects fulfilling the inclusion and exclusion criteria, who entered the study. From January 2012 and December 2019, the enrolled patients were prospectively studied.

The study procedure included four steps: (1) baseline assessment; (2) group assignment; (3) long-term care observation period; (4) follow-up assessment. In the first stage, the eligible TBI patients underwent a clinical examination at baseline. In the second stage, participants were randomly assigned to the 2 groups (LSH or telemonitoring) using a computer-generated, site-stratified, randomization schedule. Randomization was stratified according to age and sex. For each stratum, random numbers were assigned to the participants and put into envelopes; it was determined randomly whether the even or odd number would enter the LSH group. Participants were assigned to the study according to the numbers they received on opening the envelopes.

After the randomization (T0), the patients allocated in the telemonitoring group were transferred at home where they were remotely monitored by a real-time interaction service, while those assigned to the LSH group continued the medical care in the hospital. The different steps in this process were administered by different research assistants who were blinded to the other processes.

Finally, after ~4 years intervals (T1), patients from both groups were given a blind evaluation, using the same protocol as at baseline. Length of stay was extracted from charts and electronic records. This variable was defined as the time interval (in days) from the time of the discharge from IRU to the time the consultation was completed.

Caregivers enrolled in the telemonitoring in-home service underwent several training sessions. Methods used include hands-on training, staff modeling of techniques, and supervised family- led overnight stays in a transitional living apartment. Caregivers were instructed to coordinate follow-up telehealth encounters with the telemedicine center via secure messaging and to upload physiological measurements during the post-discharge phase.

### Outcome Measures

Neurological examination was performed by 2 skilled physicians who monitored the emergence of medical complications administrating the following clinical scales at admission and after the follow-up period: the Revised Coma Recovery Scale (CRS-R) (15), the Wessex Head Injury Matrix (WHIM) (16); the level of cognitive functioning as measured by the LCF (17) and the Nociception Coma Scale (NCS) (18).

### Telehealth System for Clinical Monitoring

This system is designed for patients in VS and MCS, and their families. The program is funded at an acute medical level of care to treat primary and secondary conditions and provide continuous skilled nursing (24 h/d, 7 d/wk) for monitoring all basic care activities. Patients received a monthly consultation either by a neurologist or a psychologist. Members included a physiatrist, physical therapist, respiratory therapist, occupational therapist, neuropsychologist, and family counselor. All team members are responsible for monitoring patients for signs of diminished functioning, physiological changes, infections, bedsores, new or worsening symptoms.

**Figure 1:**
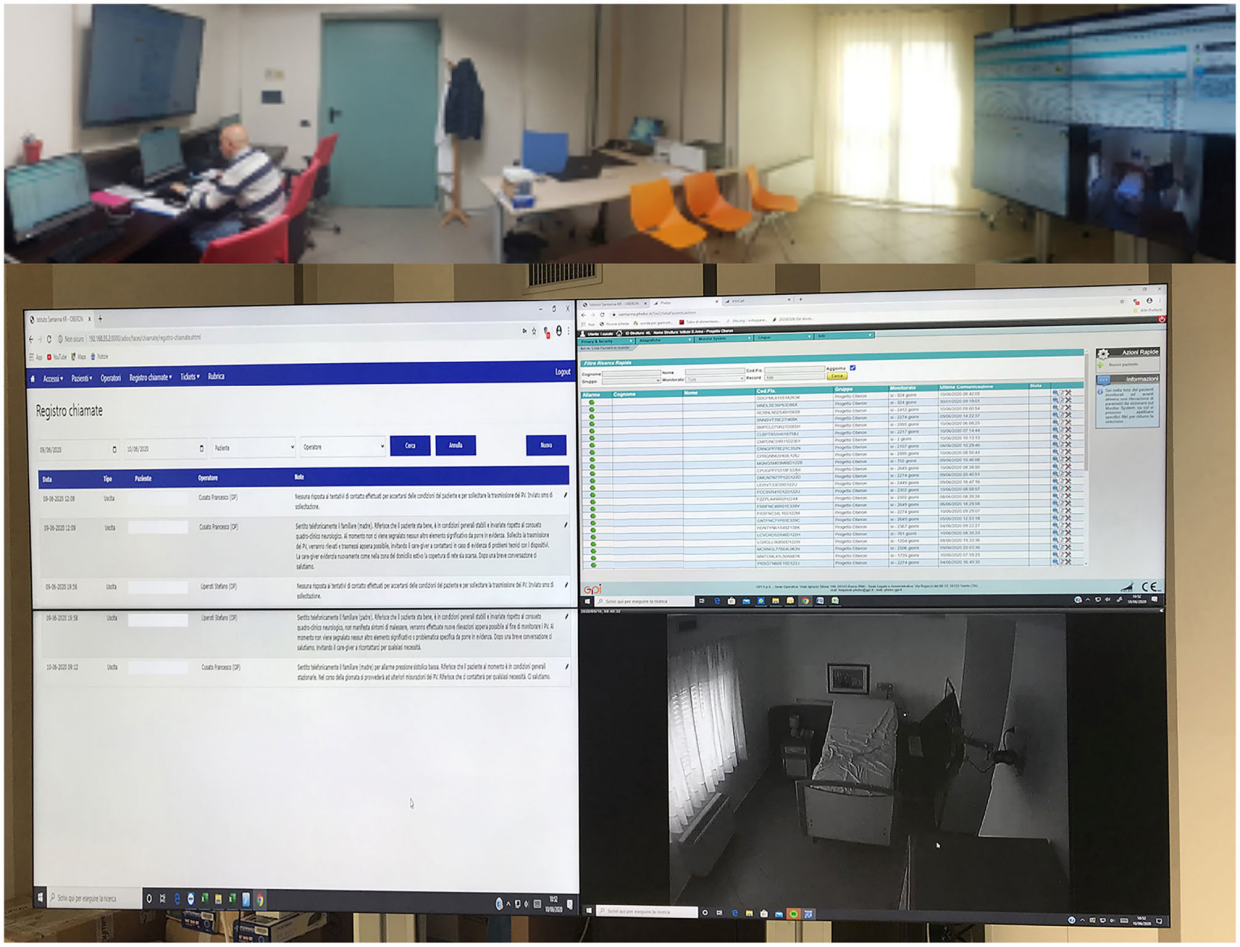
Advanced videoconferencing telehealth system for controlling neurological patients at-home.

The clinical monitoring was delivered with an advanced videoconferencing system, whereas the patients provided with low-cost monitoring devices, able to collect data about his/her health status. All treatments are based on scheduled videoconferences between the patient's home and the Clinical Units, so that the therapist can always control and modify the exercises (Figure 1). The technological device of the assistance service, dedicated to people with DOC, has been designed to manage a service center called SOU (Special Operating Unit), capable of managing real-time information generated by home and mobile workstations supplied by healthcare workers. The entire software design and architecture was built for devices running into the Android operating system, whose applications are Java-based. The Android technology was used for satisfying these particular needs:

— Operating System designed for mobile devices
— System Flexibility
— Open Source
— Kernel-based on “Linux Kernel”
— Using the Dalvik Virtual Machine to run Dalvik dex-code which is translated from “Bytecode Java” code

The solution implemented is a medical software classified as a class III (certified 93/42/EEC). The device allows us to manage video assistance services, acquisition and transmission of vital physiological parameters (e.g., pressure, glycemic rate, weight, ECG, SpO2, heart rate, etc.), questionnaires and multimedia files (e.g., image of bedsores, etc.).

The operation center has three fixed stations with diversified access for each operator registered to the system. The home workstations provide for the use of a smartphone that acts as a gateway and a series of devices connected via Bluetooth technology. App installed on the smartphone generates a visual and sound massage, at predefined intervals by the care plan, through which users are invited to measure the physiological parameters provided by the plan itself. The measurement is carried out with the devices included in the home kit delivered to the caregivers and is guided by audiovisual support through the same App.

**Figure 2:**
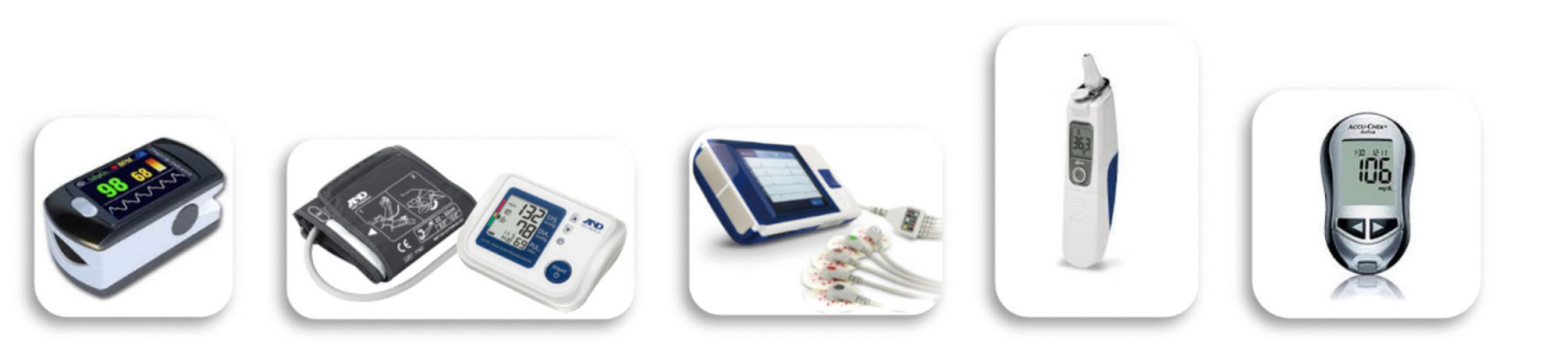
Medical devices included in the telehealth service for assessing physiological measures of patients at-home.

During the monitoring at home, the patients used wearable monitoring devices to monitor their status and to provide real-time feedback. The physiological parameters provide a measurement of the heart rate, pressure, saturimetry, temperature and glycemia (in the case of diabetic subjects) (Figure 2). These data are transmitted in real-time on the platform that compares them with the alarm thresholds defined (by an algorithm of artificial intelligence) during the creation of the medical record and based on the patient's history. The platform then returns through a color code (red/green) output to the operator present in the SOU. If an alarm occurred, the internal management procedure is activated opening the contact between the SOU and the patient's home via video call. The video call in the current home configuration is made through a portable PC and all clinical data were stored in a cloud-based system localized at S. Anna Institute for further evaluation and statistical purposes.

The entire care model is defined by 15 procedures with over 120 operating instructions and in April 2018 this obtained the UNI EN ISO 9001:2015 Certification.

The LSH program consisted of standard care focused on the treatment and prevention of secondary conditions of the primary nervous system, cardiovascular, respiratory, digestive, musculoskeletal, and skin origin. The program is adjusted in reason of the clinical needs. As for telemonitoring service, team members in the LSH program are responsible for monitoring new or worsening symptoms during the hospitalization stay period.

We also performed a cost analysis to compare our home-based, telemonitoring system to clinic-based medical assistance. The prices of all resources identified were estimated from the hospital's perspective (taking into account the proportions of public/private Italian hospitals in 2019). Costs were totaled across the 1-day treatment period and compared to clinic-based long-term assistance.

### Statistical Analysis

Statistical analysis was performed using SPSS software (version 26; Statistical Package for Social Sciences; www.spss.it). Assumptions for normality were tested for all continuous variables. Normality was tested using the Kolmogorov–Smirnov test. The analysis for differences in clinical and demographical variables at admission was made by using Chi^2^. Considering the small sample size, non-parametric statistics (Mann–Whitney U-tests and the Wilcoxon signed-rank test) were applied in order to analyze the effects of group and intervention. For all tests, a *p* < 0.05 threshold was considered to be statistically significant.

## Results

### Clinical Data

Among an initial cohort of 264 DOC patients, twenty-two TBI patients fulfilling all criteria were included in the study.

Patients enrolled in the telemonitoring program (age: 49.9 ± 20.4; 45% female; diagnosis: 36% VS; 64% MCS) were demographically and clinically matched with those admitted to LSH program (age: 55.1 ± 15; 18% female; diagnosis: 54% Vegetative State VS; 46% MCS) (Table 1). The presence of percutaneous endoscopic gastrostomy (PEG) (81 vs. 72%, in LSH or telemonitoring programs, respectively) and of tracheostomies (54 vs. 27%, in the telemonitoring and LSH programs, respectively) were similar between the two groups (Table 1).

**Table 1:** Demographic and clinical data at admission.

**Variables**	**Long-hospital stay group**	**Telemonitoring group**	***p*-level**
Number	11	11	
Sex (% female)	18%	45%	0.13^§^
Age (years)	55.1 ± 15 *51 (29–79)*	49.9 ± 20.4 *44 (21–85)*	0.39^*^
Length of stay (d)	1, 330 ± 751.7 *1,218 (418–2,968)*	1, 560 ± 805.3 *1,675 (500–2,486)*	0.12^*^
Diagnosis	54% VS 46% MCS	36% VS 64% MCS	0.39^§^
Tracheostomy (yes, %)	54%	27%	0.24^§^
PEG (yes, %)	81%	72%	0.61^§^

*Data are shown as mean ± SD and median (range). VS, Vegetative State; MCS, Minimally Conscious State; PEG, Percutaneous endoscopic gastrostomy*.

* *Mann–Whitney U-test*;

§ *Chi^2^*.

*Data expressed as median (range) are reported in italic*.

At ~4 years follow-up evaluation, patients of both groups showed similar clinical progression. Thirty-six percent of patients in the LSH program died before completing telehealth follow-up evaluation with respect to 18% of death in the other group (Table 2). Similarly, considering the evolution of medical complications during long-term chronic cure, the number of bedsores (18 vs. 0%, in the LSH or telemonitoring programs, respectively) and infections (36 vs. 18%, in the LSH or telemonitoring programs, respectively), showed a tendency to a lower number of complications in telemonitoring group, but these differences weren't significative (Table 2). Considering neuropsychological measurements, we did not find any significant difference between groups, although patients enrolled in the telemonitoring program showed better CRS-r and NCS-r scores in the follow-up period with respect to baseline (Table 3).

**Table 2:** Medical complications in TBI patients during follow-up period enrolled in the two long-term care programs.

**Variables**	**Long-hospital stay group**	**Telemonitoring group**	***p*-level**
Bedsores (yes %)	18%	0%	0.13
Infections (yes, %)	36%	18%	0.33
Death (yes, %)	36%	18%	0.33

*^§^Chi^2^*.

**Table 3:** Clinical outcome of TBI patients at admission and after follow-up.

**Variables**	**Long-hospital stay group**	**Telemonitoring group**	**Long-hospital stay group**	**Telemonitoring group**	**Statistical analysis (*****p*****-level)**			
					Between group^*^		**Within group**^**§**^	
	**Baseline**	**Baseline**	**Follow-up**	**Follow-up**	**Baseline**	**Follow-up**	**Long-hospital stay group**	**Telemonitoring group**
CRS-r	9.2 ± 3.8 *8.5 (4–16)*	10.8 ± 5.1 *10 (4–21)*	9.2 ± 5.1 *8 (4–19)*	12.4 ± 6.1 *11 (4–23)*	0.48	0.31	0.99	0.08
NCS	4.9 ± 2 *4 (3–9)*	5.8 ± 2.9 *5.5 (2–10)*	4.9 ± 2.5 *4 (2–10)*	6.3 ± 3.9 *6 (2–11)*	0.44	0.22	0.91	0.06
WHIM	20.5 ± 14.2 *18.5 (7–41)*	18.1 ± 12.2 *19 (3–53)*	22.5 ± 19.3 *17.5 (7–60)*	14.3 ± 8.4 *16 (3–25)*	0.65	0.29	0.71	0.33
LCF	2.4 ± 0.5 *2 (2–3)*	2.6 ± 0.5 *3 (2–3)*	2.6 ± 1.1 *2 (2–5)*	2.5 ± 0.5 *2.5 (2–3)*	0.35	0.81	0.31	0.99

*Data are shown as mean ± SD and median (range). CRS-r, Coma Recovery Scale-revised; NCS, Nociception Coma Scale; WHIM, Wessex Head Injury Matrix; LCF, level of cognitive functioning*.

* *Mann-Whitney U Test*.

§ *Wilcoxon W test*.

*Data expressed as median (range) are reported in italic*.

Finally, we estimated a total daily cost per patient in order to quantify the economic impact of the telehealth system with respect to hospitalization. The mean total cost per patient in the LSH group was 262€, whereas in the telemonitoring group cost was approximately 93€. The different components of cost are shown in Table 4. The major component of cost for the LSH program was human resources focused on staff time dedicated to patient care, whereas for telehealth program approximately half of the health costs relied on the equipment (i.e., medical devices) (Table 4).

**Table 4:** Components of daily health care costs.

**Sub-components**	**Long-hospital stay group**	**Telemonitoring group**
Nursing and Staff time (€)	116	25
Medication (€)	23	10
Hospitality (€)	90	0
Equipment (€)	30	48
Internet Connection (€)	0	0.5
Transfers (€)	0	5
Caregivers Training (€)	3	4.5
Total (€)	262	93

## Discussion

In this study, we provide preliminary evidence about a new telehealth service useful to monitor patients with DOC, secondary to TBI. Overall, we showed that in a wide temporal window (4 years), the clinical condition of disease was similar in a group of demographically and clinically-matched patients admitted in a traditional LSH program with respect to telemonitoring service. Indeed, we found the maintenance of a stable and similar: (a) cognitive status (as measured by the CRS-r and LCF scales); (b) level of responsiveness to the environment (i.e., pain stimulation) (as measured by the WHIM and NCS scales); and (c) occurrence of medical complications (i.e., bedsores, infections). Moreover, our data confirm the cost-effectiveness of our system, since we found that delivering assistance by telemonitoring is less expensive than providing the same service in the hospital.

Despite no significant difference was detected in all clinical evaluations, it could be highlighted that patients telemonitored at home showed a trend toward a better clinical picture (Table 3). Our telemonitoring service allows us to organize remote treatments by means of videoconferences made by the clinicians with the caregiver/family, answering every question about the clinical condition, observing the progression of medical complications and suggesting how to prevent them. This service is useful to guide caregivers in different steps of treatment relative, for instance, to the management of complex medical complications, such as tracheostomy and route of feeding, as well as bedsores. Furthermore, the daily monitoring of vital signs and the phone contact between caregivers and the telehealth operators allowed the family to consider their relatives involved in a “protected” room like the hospital, avoiding feelings of abandonment and stress (19, 20). In this way, caregivers act with a more consistent role in monitoring the outcomes of their relatives (21).

Our data confirm the potential of telehealth for the chronic management of TBI patients. As already demonstrated for other remote delivery systems proposed for elderly (8), PD or AD populations (11, 22), the feeling of being followed and cared at home plays a key role also in the clinical progression of severe TBI patients. Considering the recent statement of WFNR for telerehabilitation (http://wfnr.co.uk/), evidence on the effectiveness of telecounselling concluded that providing support to family members of people with TBI was beneficial (22) and that telecare is accepted by the vast majority of TBI patients and their careers (23). Studies on the effectiveness of the tele-based therapy in comparison with outcomes reached during usual LSH demonstrated significant improvements in global functioning, sleep quality, and depressive symptoms (24). Taking together all these findings we can conclude that telerehabilitation is as efficacious as usual in-person care for individuals with TBI (24, 25). However, considering the telemonitoring level, there is a paucity of data. For this reason, we believe that our preliminary study has the potential to increase the relevance of this kind of technology for the management of TBI patients. This is very important, considering that the main risk of TBI-related rehabilitation is that the recovery achieved in the hospital will be lost at home.

### Limitations

Some limitations of this study need to be discussed. Firstly, our sample size was relatively small. However, it should bear in mind that to avoid spurious data coming from the well-known clinical heterogeneity characterizing TBI highly stringent inclusion criteria have been employed. Despite a perfect matching in demographical and clinical variables between groups at baseline, we recognized that a more exhaustive evaluation of medical complications during the follow-up period should be performed in further studies. Second, telemonitoring at home is generally more suitable for TBI patients with a stable clinical condition. Finally, the telerehabilitation technology is not always viewed by caregivers as a common practice, which can often require frequent consultations to SOU, causing a significant burden, especially in remote locations. Our system is considered feasible and accepted by all patients although evidence by satisfaction questionnaires was not provided since we are completing it.

## Conclusions

Considering a mean follow-up period of approximately 4 years, we demonstrate that our telehealthcare service provides similar performance, as in-person usual care, to manage a complex neurological disorder such as TBI. As with other well-known telemonitoring programs based on a patient-centered approach to care, we also demonstrate that this kind of patient might be followed outside of the hospital in a cost-effective way, although deeper quantifications of direct/indirect costs are preferred. Further studies including different etiologies (i.e., vascular, anoxic) are needed to better define the limits of telehealth in DOC patients and to guide the policy decisions about the systematic use in health care (26). However, it is mandatory to translate the feasibility and acceptability of this kind of telemedicine platform from neurological patients to other clinical domains. As recently stated by Maresca et al. (27), telemedicine services will contribute to a transformation of the entire healthcare sector and business models, mainly in the era of pandemics (i.e., COVID-19), where there is a need to avoid direct contact between clinicians and patients and to reduce the number of admissions at hospital.

## Data Availability Statement

The raw data supporting the conclusions of this article will be made available by the authors, without undue reservation.

## Ethics Statement

The studies involving human participants were reviewed and approved by Central Area Regione Calabria, Catanzaro, Italy. The patients/participants provided their written informed consent to participate in this study.

## Author Contributions

Statistical analysis was done by SL, LM, and AC. The study design was done by LP, PT, and AC. Drafting the manuscript was done by AC, AM, and FC. Clinical data collection was made by FA, MGR, LR, and SS. Literature search, data interpretation, and revising the manuscript were done by FA, LP, PT, and AC. All authors contributed to the article and approved the submitted version.

## Conflict of Interest

The authors declare that the research was conducted in the absence of any commercial or financial relationships that could be construed as a potential conflict of interest.
